# A Rust Extraction and Evaluation Method for Navigation Buoys Based on Improved U-Net and Hue, Saturation, and Value

**DOI:** 10.3390/s23218670

**Published:** 2023-10-24

**Authors:** Shunan Hu, Haiyan Duan, Jiansen Zhao, Hailiang Zhao

**Affiliations:** 1 School of Automotive Engineering, Changshu Institute of Technology, Changshu 215506, China; 2 Merchant Marine College, Shanghai Maritime University, Shanghai 201306, China

**Keywords:** rust extraction, navigation buoy, improved U-Net method, HSV

## Abstract

Abnormalities of navigation buoys include tilting, rusting, breaking, etc. Realizing automatic extraction and evaluation of rust on buoys is of great significance for maritime supervision. Severe rust may cause damage to the buoy itself. Therefore, a lightweight method based on machine vision is proposed for extracting and evaluating the rust of the buoy. The method integrates image segmentation and processing. Firstly, image segmentation technology is used to extract the metal part of the buoy based on an improved U-Net. Secondly, the RGB image is converted into an HSV image by preprocessing, and the transformation law of HSV channel color value is analyzed to obtain the best segmentation threshold and then the pixels of the rusted and the metal parts can be extracted. Finally, the rust ratio of the buoy is calculated to evaluate the rust level of the buoy. Results show that both the segmentation precision and recall are above 0.95, and the accuracy is nearly 1.00. Compared with the rust evaluation algorithm directly using the image processing method, the accuracy and processing speed of rust grade evaluation are greatly improved.

## 1. Introduction

Navigation buoys, also known as navigational aids, are facilities with information service functions used to ensure ship safety, economy, and navigation convenience. For the navigation buoy system, there are many and widely distributed buoys [1], which are often affected by many factors, such as wind, waves, climate, or impacts, that result in abnormal conditions; significant tilting or severe corrosion may cause damage to the buoys themselves. Timely extraction of abnormal buoys and taking corresponding repair measures are important means to ensure the safety of ships’ navigation.

Installing a telemetry and remote-control module on the buoys [2] not only can transmit the information from the underwater buoy(s) to the management personnel’s computer but can also collect basic information such as the position and light address of the navigation buoy. However, basic information such as the position and light address of the buoys can only be collected, and information such as the structure and coloring of the buoys cannot be obtained. Poor rust resistance and stability exist in the buoy system. In complex navigation environments, the buoy body, layers, etc., are easily damaged by external adverse factors, resulting in safety hazards for ship navigation. The degradation of steel infrastructure is most obvious in the form of rusting, and rust removal is an important step to maintain the safety of facilities. As an important aid to navigation, navigation buoys float on the water surface for a long time and are distributed in various waters and seas. Under the influence of an abusive environment, the metal part of the coating of navigation buoys will fall off and the metal surface is exposed to an oxidation reaction with oxygen and water in the air, generating ferric oxide (${\mathrm{Fe}}_{2}O_{3}$), i.e., rust. Rust is the collective name for iron oxides, which usually present a red or reddish-brown color.

With the development of computer vision technology, object detection has become one of the research hotspots in the field of computer vision [3,4] and has received widespread attention in fields such as power inspection [5] and road traffic [6]. In the field of navigation, the recognition of targets such as ships and sea skylines is the focus of research [7,8], but there is relatively little research on the detection of navigation aids. Unmanned equipment, such as drones and unmanned ships equipped with visual sensors, is used to obtain image data during the inspection. By using machine vision-related technologies to detect anomalies in the buoys, inspection work can be completed under different environmental conditions. It greatly improves the efficiency of buoys’ inspection, reduces the cost of navigation aids’ inspection, and enhances the automation and intelligence level of buoys’ inspection.

Color model conversion methods, such as HIS, RGB, HSV, YCrCb, etc., are used in traditional image segmentation systems; these methods select the color space model and color components with the most significant rust color features relative to the background area, and perform image segmentation to achieve rust detection and extraction. Choi and Kim [9] classified five types of rust using hue, saturation, and intensity (HIS) color spaces and varimax methods as well as statistical analysis of red, green, and blue (RGB) color spaces. By conducting statistical analysis on the color spaces of red, green, and blue (RGB), Lee et al. [10] were able to detect small rust buoys on steel bridge coatings relative to background information. Ghanta et al. [11] used the concepts of pattern recognition and Haar wavelet transforms to detect rust in RGB sub images of steel coated bridge surfaces. Zhang et al. [12] converted aerial photographs of high-voltage transmission line corrosion images from RGB space to YCrCb space models. The experimental results showed that the corrosion feature extraction algorithm can identify rust areas on transmission lines, which can improve the efficiency of transmission line maintenance.

Deep learning methods, such as convolutional neural networks (CNN) and recurrent neural networks (RNN), are used to detect rust on metal surfaces [13]. Du J et al. [14] proposed an improved CNN model based on corrosion images to classify and evaluate the degree of corrosion in grounding grids. Yao et al. [15] trained a large number of corrosion images through the CNN to obtain the classifier model, and then used the classifier model and overlapping scanning sliding window algorithm to identify and locate the corrosion of the hull structural plate. Forkan ARM et al. [16] proposed a CorrDetector framework based on deep learning method, which uses CNN as the basis for structural identification and corrosion feature extraction and is used for corrosion detection of complex scenes such as telecommunications towers photographed by UAVs. 

Traditional image segmentation detection methods can use the color features of rust to extract all areas that match the rust color [17]. However, there are some misjudgments in these methods. The background areas may be misjudged with similar colors to the rusted areas. The rusted region segmentation model based on deep learning has achieved certain results, with the advantages of fast detection speed and high segmentation accuracy [18]. However, the complex and ever-changing image background makes it difficult for the model to fully focus on rust features, so there are still many problems in the engineering practice of rust detection and segmentation that urgently need to be solved. However, due to the unique nature of the scene, there are problems with light reflection from the water and the buoys are always swaying. At present, the rust evaluation method for the buoys is not developed enough. 

Some researchers use improved U-Net methods to achieve segmentation. Compared with traditional CNN, U-Net has fewer training images and better performance [19], but this model has shallow layers, fewer parameters, and poor real-time performance. Therefore, many improved U-Net methods have emerged. Shi et al. [20] studied the impact of two dataset construction methods, compression segmentation and crop segmentation, on the performance of steel bridge rust segmentation based on the VGG U-Net. Jiang et al. [21] proposed an improved algorithm FAU-Net for the internal scene of concealed steel box girders, which embeds fusion modules and attention modules in the network for aggregating multi-level features and learning feature information, respectively. An end-to-end U-Net-based segmentation framework named DA-Net was proposed by Maqsood et al. [22] for efficient lung nodule segmentation. 

Therefore, an innovative rust detection and evaluation method for maritime buoys is proposed. The method includes an improved U-Net and HSV. The improved network combines a residual network and a squeeze-and-excitation (SE) attention module to increase the segmentation accuracy and processing efficiency. Then, the image is converted from RGB space to HSV space by a nonlinear transformation formula to extract rust areas more accurately. Finally, the ratio between the area of the rusted and the metal parts is calculated to give the rusted level of the buoy. The contribution of this article is twofold: the first is that we improve the segmentation accuracy and rust extraction effect under the condition of a small number of datasets and the second is that we provide an evaluation level of rust of the buoys.

The rest of the paper is as follows: Section 2 is the method, Section 3 is the experiment, and Section 4 is the analysis of the results.

## 2. Theoretical Method

To achieve fast and high-precision rust detection and evaluation under the condition of a small number of datasets. The improved U-Net and HSV color spaces are used.

### 2.1. U-Net Model

**Figure 1:**
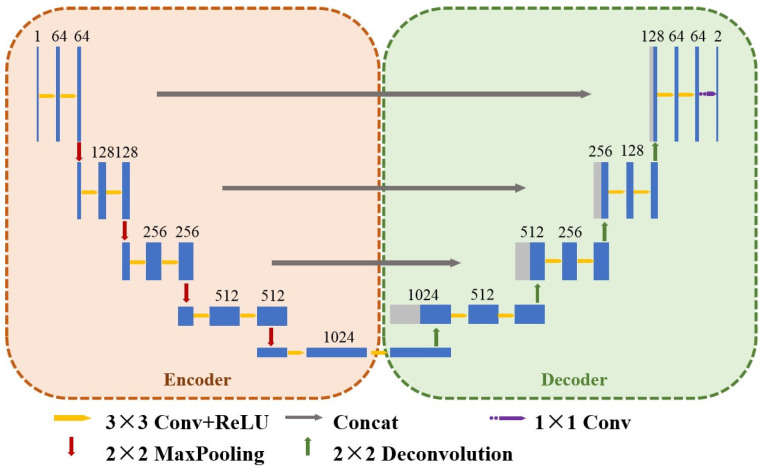
Conventional U-Net structure.

The conventional structure of U-Net [23] model (shown in Figure 1) mainly consists of three parts: encoder, decoder, and jump connection, which extracts deeper feature information by downsampling operation in the encoder, and extracts lower-level features and restores image detail information and resolution by upsampling in the decoder. Multiple jump connections are used to connect the corresponding features between the encoder and decoder and to help the decoder to better restore image detail information and resolution by fusing the corresponding shallow and deep features. In the encoder part, the input image is successively downsampled through four sets of coding blocks to extract features, each of which contains two convolutional layers with 3 × 3 convolutional kernels: one ReLU activation function layer and one 2 × 2 maximum pooling layer. 

For the network, the mapping function of the model is as follows:

Y = F(X, W)(1)

where F( ) is the network model, X is the input image, Y is the segmentation result, and W is the weight of the network. The input image is a grayscale image. In a traditional convolutional layer, the dilated rate supposed by us is r, the kernel size of convolutional filter is k, so the receptive field R_k_ is as follows:

R_K_ = (k − 1) × (r − 1) + k (2)

### 2.2. Improved U-Net Model

**Figure 2:**
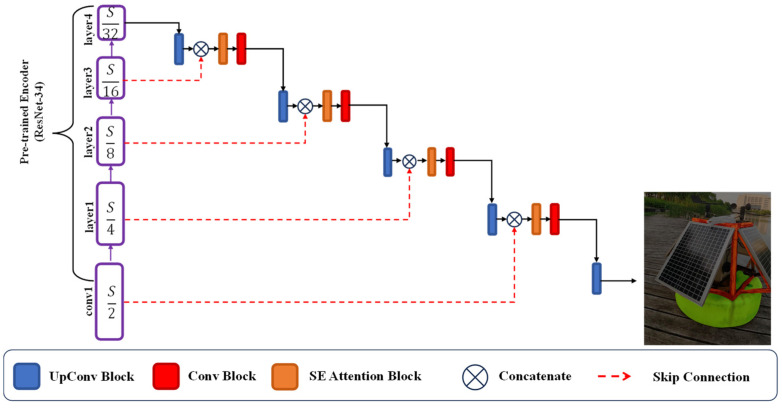
The proposed network framework.

For the rust area segmentation task, the segmentation results can be affected by the effectiveness of the features extracted from the input image. To enhance the extraction capability of the network, a ResNet [24] with the final pooling and fully connected layers removed is adopted. Richer features can be effectively obtained because of the deeper network and less FLOPs. The improved network is based on the U-Net. The encoder–decoder architecture and equal amounts of downsampling and upsampling segmentation network are adopted, shown in Figure 2. The transfer learning methods can be introduced with the help of the proposed framework, which solves the problem of insufficient dataset. To solve the problem of weak computational capability, an SE attention module is introduced to the decoder. The interdependence between channels is set up to guarantee its performance. In Figure 2, S represents the spatial resolution of the input image. For corroded and non-corroded areas, two input feature maps with different resolutions for each layer in the decoder are used. The corroded feature is sampled by the UpConv block. The SE attention block is utilized to reflect the relationship between channels. It is also used for solving the disadvantage of low FLOPs. 

The part of segmentation is pixel classification, so the cross-entropy loss function to supervise the training of the network, which can be expressed as follows:

(3) $$L_{dice}\left(X,Y\right)=1-\sum_{k=0}^{K}\frac{2\omega_{k}\sum_{i=1}^{H}\sum_{j=1}^{W}P\left(X\left(i,j\right),k\right)g\left(Y\left(i.j\right),k\right)}{\sum_{i=1}^{H}\sum_{j=1}^{W}p\left(X\left(i,j\right),k\right)+\sum_{i=1}^{H}\sum_{j=1}^{W}g\left(Y\left(i.j\right),k\right)}$$

where *H* and *W* are the height and width of the image; *K* is the number of categories except the background; *g*( ) is the truth label for every *K*. 

The additional computation of the module is almost negligible. The architectural details of the encoder and decoder of the network are shown in Table 1. 

### 2.3. HSV Color Space

**Figure 3:**
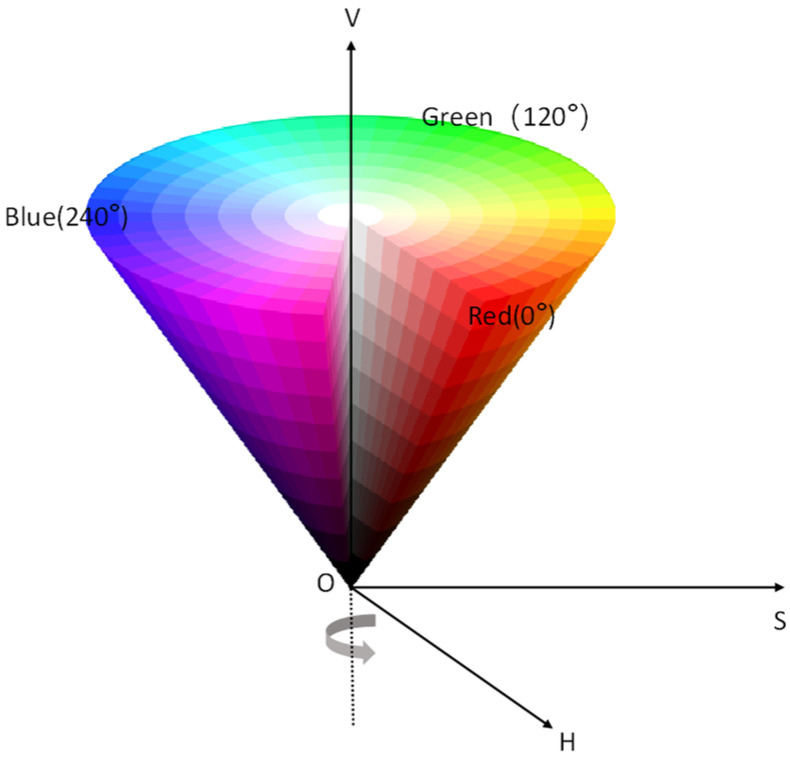
HSV color model.

Machine vision-based image processing methods usually convert color images into grayscale images, and screen oxidation layers and contaminated areas by grayscale thresholds [25,26,27]. However, for the rusted surface of metal material, it is difficult to identify the rusted area because the gray value of the rusted area in the grayscale image is very close to the gray value of the metal material substrate and surface oil, etc. Therefore, this paper adopts a color space conversion algorithm to convert RGB images into HSV space, realizes the extraction of rusted areas on steel surfaces by setting extraction thresholds, and calculates the percentage of rusted parts to the total pixel points of the image to determine the degree of rusting. HSV (shown in Figure 3) is a color space model established in a cylindrical coordinate system, as shown below. Where H is hue, S is saturation, and V is luminance, where the value range of hue H is [0°, 360°], and red, green, and blue are 120° away from each other; the value range of saturation S is 0–1, and only grayscale when S is equal to 0; the value range of luminance V is the same as saturation S, which is not directly related to light intensity. The RGB of the color image is exchanged to HSV space by the nonlinear transformation formula.

(4) $$H=\left\{\begin{array}{l} 0\;\;\;\;\;\;\;\;\;\;\;\;\;\;\;\;\;\;\;\;\;\;\;\;\;\;\;\;\;\;\;\;\;\;\;P_{max}=P_{min}\\ 60\times \frac{g-b}{P_{max}-P_{min}}+0,\;\;\;\;\;P_{max}=r\;\mathrm{and}\;g\geq b\\ 60\times \frac{g-b}{P_{max}-P_{min}}+120,\;P_{max}=r\;\mathrm{and}\;g\leq b\\ 60\times \frac{g-b}{P_{max}-P_{min}}+240,\;P_{max}=g\;\;\;\;\;\;\;\;\;\;\;\;\;\;\;\;\;\\ 60\times \frac{g-b}{P_{max}-P_{min}}+360,\;P_{max}=b\;\;\;\;\;\;\;\;\;\;\;\;\;\;\;\; \end{array}\right.$$

(5) $$S=\left\{\begin{array}{l} 0\;\;\;\;\;\;\;\;\;\;\;\;\;\;\;\;\;\;\;\;\;\;\;\;\;\;\;\;\;\;\;\;\;\;\;\;P_{max}=0\\ \frac{P_{max}-P_{min}}{P_{max}}=1-\frac{P_{min}}{P_{max}},\;P_{max}\neq 0 \end{array}\right.$$

V = P_max_ (6)

where r, g, and b denote the chromaticity values of red, green, and blue in RGB images, respectively, $P_{\max}$ and $P_{\min}$ denote the extreme and minimal values of the three chromaticity values of r, g, and b, respectively.

## 3. Experiment

**Figure 4:**
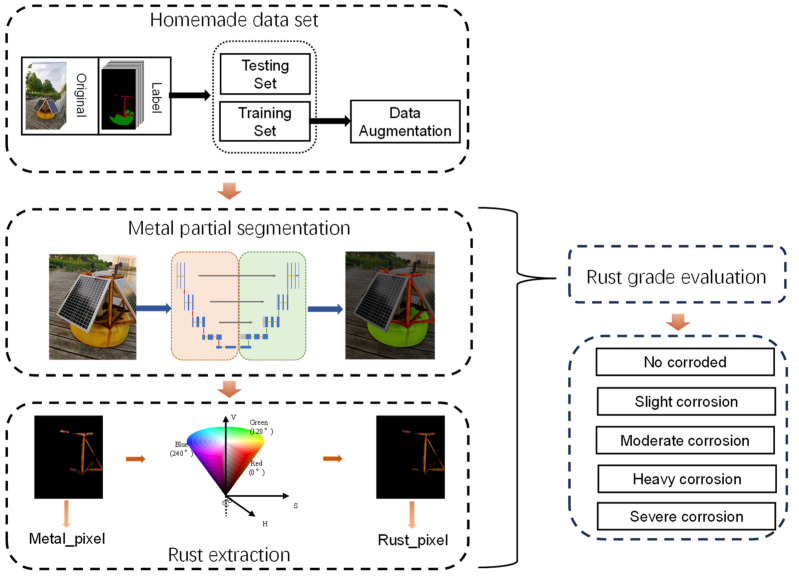
Flowchart of the experiment.

The experimental process is shown in Figure 4, it includes dataset establishment, image segmentation, rust extraction, and rust evaluation. We use the image segmentation method to segment the metallic areas of the entire image, and then extract the rust from the metallic parts. For navigation buoys metal partial segmentation, due to the limitation of rust detection dataset and SegNet [28] and DeepLab [29] networks are for natural image domain with large semantic segmentation dataset, but conventional U-Net is initially applied to medical image segmentation domain with small sample dataset. The experimental procedure is shown in Figure 4, we use an improved U-Net as the backbone network and initialize the parameters with ResNet-34 [23] pre-training weights to achieve segmentation of navigation buoy images. 

The advantages of the proposed improved U-Net in the experiment are less data and lower requirement for computer configuration. Then, the transformation law of HSV channel color value is analyzed to obtain the best segmentation threshold. 

### 3.1. Evaluation Metrics

After using the rust detection model, the total number of pixels (*metal_pixel*) in the metal part, and the total number of corroded pixels (*Rust_pixel*) in the metal part can be output.

(7) $$r_{Rust}=\frac{Rust\_pixel}{Metal\_pixel}$$

This evaluation method is based on the Chinese national standard GB/T8923-2011 [30] for evaluating corrosion defects on steel and metal surfaces. We evaluate the corrosion of navigation buoys based on different corrosion ratios; Table 2 shows the relevant evaluation for corrosion level.

Precision (P), recall (R), and average precision (AP) are often used in target detection to measure the performance of target detection algorithms. Precision (precision) refers to the accuracy of target detection, recall (recall) refers to the ratio of the number of true targets detected to the number of all targets detected, and average precision (AP) is the area below the P-R curve for a single target class.

(8) $$\mathrm{Precision}=\frac{TP}{TP+FP}$$

(9) $$\mathrm{Recall}=\frac{TP}{TP+FN}$$

where TP (true positive) refers to the number of pixels that are actually true and predicted to be true, FP (false positive) refers to the number of pixels that are actually false but are predicted to be true, and FN (false negative) refers to the number of pixels incorrectly predicted as counterexamples. The F1 score depends on recall and accuracy:

(10) $$F1=2\times \frac{Recall\times Precision}{Recall+Precision}$$

Accuracy (Acc): accuracy is the ratio of the number of all correctly classified pixels to the total number of pixels and is expressed as follows:

(11) $$\mathrm{Acc}=\frac{TP+TN}{TP+TN+FN+FP}$$

Intersection and merge ratio (IOU): a metric used to measure the similarity between labeled and predicted images. It treats the labeled image and the predicted image as two sets and calculates the ratio between their intersection and merge sets. The expression of IOU is as follows:

(12) $$\mathrm{IOU}=\frac{TP}{TP+FP+FN}$$

### 3.2. Experimental Environment and Configuration

The experiments are conducted on Windows 10 with NVIDIA Geforce RTX 2070 GPU, Inter(R) Core(TM) i7-11800H CPU, and 16 GB RAM. The development environment is as follows: integrated development environment is PyCharm2020.3.5, programming language is Python 3.8.11, deep learning framework is PyTorch1.8.1. The reason for selecting these configurations is that the proposed and comparative algorithms can run smoothly and quickly in these conditions. The experimental environment is selected during the day to ensure illumination. During the filming process, we choose weather with good visibility and calm winds and waves. The camera is shot parallel to the horizontal plane, which greatly reduces the reflection of the water surface. 

In order to speed up the network model training time, this experiment divides the training process into two phases. Since convolutional neural networks share feature extractors, performing freeze training helps to speed up the model training. In addition, the migration learning idea is introduced in this experiment for faster convergence. During the training process, the number of rounds is denoted as epoch, and one epoch represents a complete training of the model using one dataset. Considering the influence of objective factors such as actual equipment hardware, the batch size is set to 1, i.e., one sample is selected for training at each training session. Different batch sizes will generate varying degrees of impact on the learning of the network model. A total of 300 training rounds are conducted, freeze training is used in the first 100 rounds, while thaw training is used in the 101–300 rounds. Meanwhile, the learning rate weight decay value is set to 0.00005, which can reduce the risk of overfitting the network and help achieve better generalization performance.

Information on the configuration of the parameters used during the experimental training of the U-Net-based metal partitioning algorithm is shown in Table 3 as follows:

### 3.3. Dataset Production

**Figure 5:**
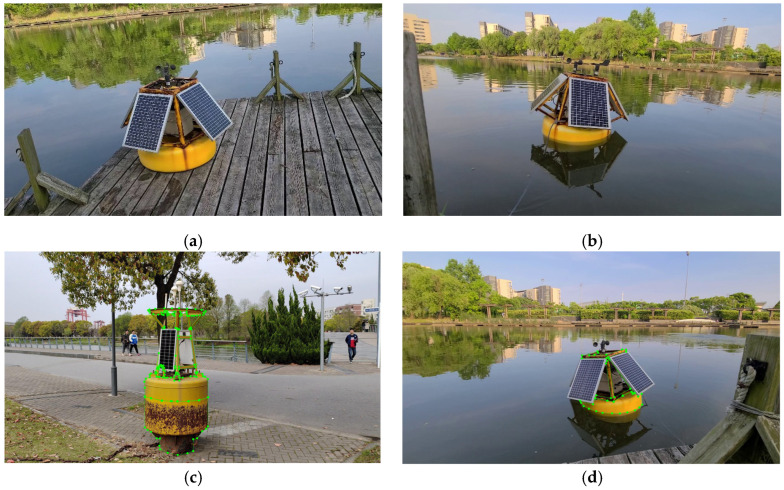
Sample dataset for rust detection of the buoys. (**a**,**b**) Sample dataset, (**c**,**d**) Sample dataset labelling, the green dots are used to mark the outline of the buoy.

Thirty of these images with rust are selected as the dataset, which contains both land-based and water-based scenes; this paper uses the same equipment for another rust-free beacon to collect the dataset, and 30 images without rust were selected. Parts of the dataset are shown in Figure 5a,b. The camera used to capture the images is MV-CA050-11UM, and the resolution of the images is 3840 × 2160. The pixels of the images are 1080 × 1920. The lighting conditions are very good, and there is basically no reflection on the water surface. In summary, the self-constructed dataset of beacon rusting consists of 200 images of beacons. The open source annotation tool LabelMe is used to manually label (Figure 5c,d) the metal areas of the collected images, and the labeled images are eventually converted from JSON format to PNG format for preservation. To solve the problem of low data volume, data enhancement methods such as flipping, random rotation, and filling are used to improve the generalization and robustness of the network. All the input images are rescaled to between 35% and 150% of their original resolution, then they are horizontally vertically, and diagonally, flipped with the same probability of 33.3%. Finally, the hue, saturation, and value are randomly adjusted to between 50% and 150% of the original value. The final data volume is increased to 500 images. We split the original dataset into 70% for training and 30% for testing, then the data augmentation is performed. In the process of data augmentation, we only enhance the training set.

## 4. Results and Analysis

### 4.1. Segmentation of Metal Parts

**Figure 6:**
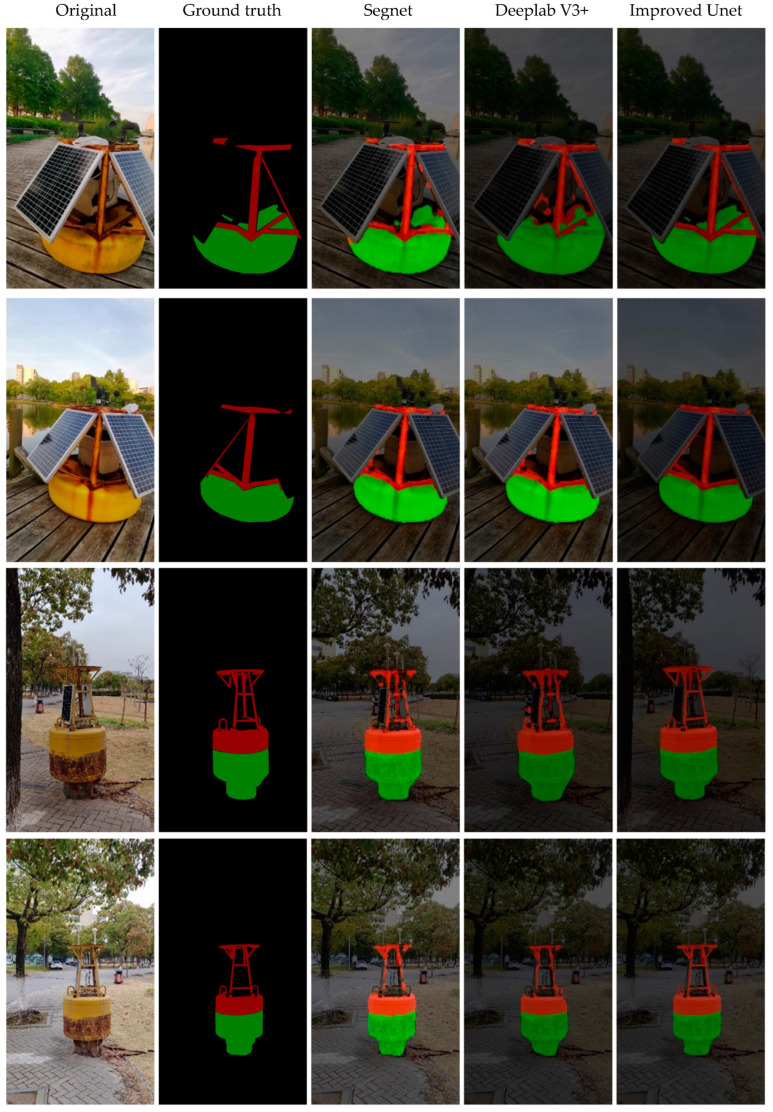
Comparison results of metal part segmentation using different models. For the ground truth, the red part represents metal, the green part represents the segmented pontoon, and the black part represents the background.

The image processing-based method can only extract the rust in a small range of specific areas, and the framed images of specific areas also contain the remaining non-metallic parts of the beacon, and the rust level evaluation is not accurate, so the improved U-Net method is used in this study to segment the metal areas of the buoy in the whole image, and then extract the rust from the metal parts. In order to verify the effectiveness of the metal part segmentation model in the rust detection method constructed in this study, the deep learning-based semantic segmentation model SegNet, DeepLab V3+ [31], were used as a comparison test to segment the metal regions on 30 test sets in the constructed dataset under the same environment, and the segmentation results of some of the test images are shown in Figure 6. From the results of segmentation using three different image segmentation algorithms for the metal part of the buoy are shown, and it can be seen that the segmentation effect of the improved U-Net is better and can achieve more accurate extraction for the metal part of the beacon. For the SegNet model, all three images have more false detection and missed detection. In Figure 6, the red zone is the segmented metal part, the green zone is the segmented pontoon part, and the black zone is the background. 

Table 4 shows the comparison of evaluation indexes of the five different segmentation models for testing data. In addition to the model we proposed, four state-of-the-art methods (the latest versions of SegNet and DeepLab as well as PSP Net [32] and DA Net [22]) are adopted. Combined with Figure 6, other models in the experiment have more serious leakage extraction due to the small amount of data in the rust segmentation dataset; their IOU values are no high than 0.91, and the U-Net model used in this paper has better indexes of all kinds than the other four models. For example, the F1 score is nearly 0.98, the accuracy reaches nearly 1. This proposed method can achieve excellent data processing speed under the condition of a small number of data sources.

Since the problem to be solved in this study is the accurate extraction of metal parts, the high-accuracy segmentation of single category is especially important. We want to investigate the effectiveness of evaluation algorithms for segmentation tasks to choose the best method. Table 5 shows the comparison of evaluation indexes of five different algorithms for single-category (metal) segmentation for testing data. It can be seen that the improved U-Net outperforms SegNet and DeepLabV3+ models in all indexes for metal category segmentation, among which, precision is 35.3% higher than for the SegNet model, 15.7% higher than for the DeepLabV3+ model, 10.9% higher than for the PSP Net model and 6.7% higher than for the DA Net model; recall (recall) is 3.7% higher than for the SegNet model, 6.6% higher than for the DeepLabV3+ model, 8.4% higher than for the PSP Net model, and close to that of the DA Net model; accuracy (Acc) is close to 1; and intersection ratio (IOU) is 33.2% higher than for the SegNet model, 18.2% higher than for the DeepLabV3+ model, 11.6% higher than for the PSP Net model, and 5.3% higher than for the DA Net model. However, from Table 6 and Table 7, it can be seen that the processing speed of our improved U-Net is faster than other models. It can also be seen that for non-improved U-Net method, the processing speed is relatively slow.

To demonstrate the effectiveness of data augmentation. Table 6 shows the comparison with and without data augmentation. It can be seen that all the parameters, precision, recall, Acc, and MioU, are improved. The effectiveness of data augmentation is verified.

### 4.2. Extraction and Assessment of the Rust

**Figure 7:**
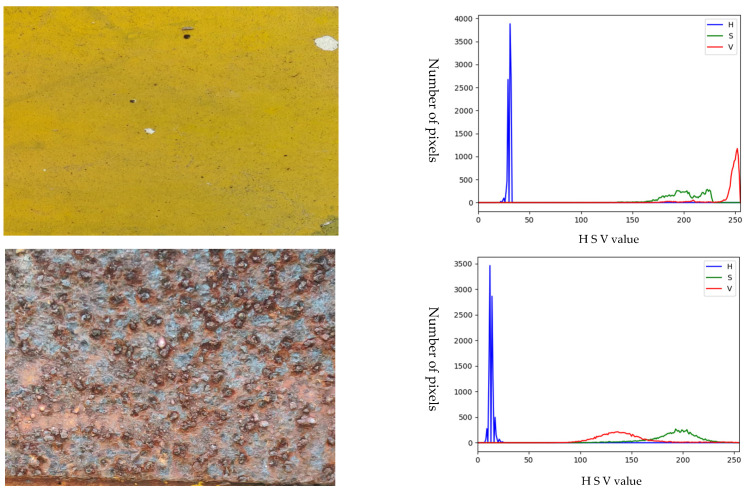
Color histogram of rusted and rust-free images.

HSV color histograms with and without rust are shown in Figure 7, from which a range of HSV color values can be obtained. It is clear that the HSV curves with and without rust are quite different. It is worth mentioning that the rust may exist in the non-metal parts, which can affect the accuracy of rust extraction and assessment. Therefore, the method of image segmentation is used to extract the metallic parts while filtering out the non-metallic parts to improve the accuracy of rust extraction.

HSV color thresholds for rusted objects on metal surfaces are shown in Table 7. It is worth mentioning that in order to facilitate equation programming, the thresholds for H, S, and V used here are based on the HSV numerical range of OpenCV. So here, the chromaticity H takes the value range from 0 to 15, the saturation S takes the value range from 140 to 255, and the luminance V takes the value range from 80 to 180. First, the original image is converted to HSV format to obtain the three components of H, S and V for each pixel point. Create an image with a black background, the same size as the original image. Then all the pixel points in the original image are traversed, and if the H, S, and V components of the pixel point are within the set threshold range, respectively, the pixel point is copied to the black background. The final image obtained is the image composed of the pixel points that meet the HSV color threshold requirements. 

**Figure 8:**
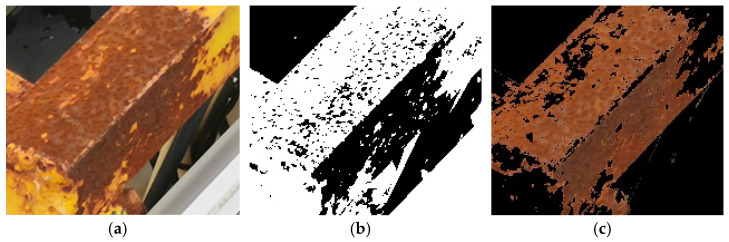
Comparison of extracted rusts based on grayscale threshold and HSV color space conversion. (**a**) Area to be tested, (**b**) grayscale threshold image, and (**c**) HSV color space conversion image.

Figure 8 shows the comparison of the results based on the extraction of rust on the surface of the buoy by two different methods, the one processed by the grayscale threshold method is shown in Figure 8b, and the result of HSV color space conversion is shown in Figure 8c. The grayscale threshold is set to 90, and the pixel points above this setting will be transformed into white, and the rest of the pixel points will be transformed into black, with black representing the rusted part. By comparison, it can be seen that the results extracted by the grayscale threshold method are not accurate. The black color of the area to be detected is not accurate:

1. The black part of the area to be detected will be detected by mistake;
2. The rusted area is not fully extracted, and the lightly corroded part is not extracted.

By comparing the results of the grayscale threshold-based rust extraction method with those of the HSV color space conversion method, it can be seen that the former method has the problem of partial extraction of non-rusted objects and incomplete extraction of some rusted areas; the latter, on the other hand, is able to capture the rusted part of the object in the image well, which fundamentally solves the deficiency of the gray-scale threshold method in the accuracy of steel rusted objects detection. Therefore, the HSV color space conversion method can significantly improve the detection quality of buoy corrosion.

In order to indicate the degree of rusting of the buoy, this study assessed the rusting of the metal part of the buoy based on the ratio value of the pixel value of the rusted area to the total pixel value of the metal area image and used the calculation Equation (7) to obtain the rust ratio and rust grade.

**Figure 9:**
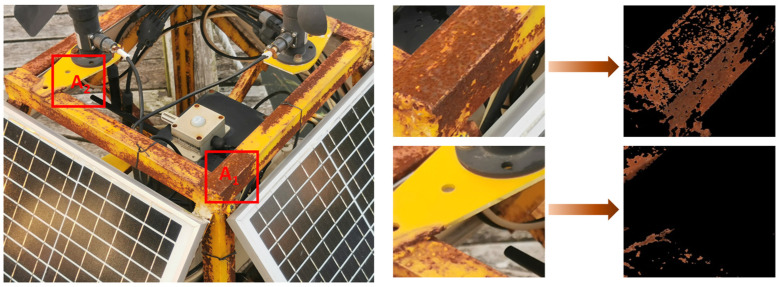
Comparison of rust extraction results for some areas of the buoy.

We select two regions from the same buoy and treat them as A_1_ and A_2_. As shown in Figure 9, the comparison table of pixels and ratio of the two areas is shown in Table 4. A_2_ area has a small amount of rust, as shown in Table 8, the counted rust pixel value is 1832, accounting for 7.17%, and the detection result is slight rust; meanwhile, the rusted part of A_1_ rust area accounts for 77.82%, which is extremely severe rust.

**Figure 10:**
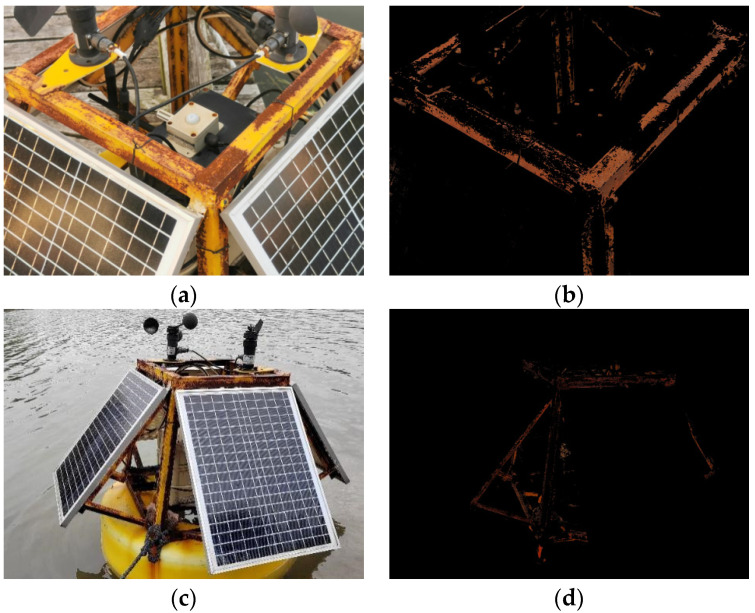
Rust extraction results based on color space conversion. (**a**,**c**) are the original figures, (**b**,**d**) are the rust extraction.

Figure 10 shows the experimental results of rust extraction of buoys; Figure 10a: the image has the influence of light; and Figure 10c: the image has the influence of water surface. Figure 10b,d, respectively, are two graphs of rust extraction results based on the color space conversion method, both can extract the rust area more accurately. 

The comparisons of pixels and ratios are shown in Table 9. Under the influence of light, there may be false positives. The original pixels of the visible image include navigation buoys and other environmental parts, so there is a significant error in calculating the proportion of pixels in the corroded part. In summary, the non-metallic part of the buoy has a greater impact on the detection of rust and the assessment of the rust level of the beacon. 

**Figure 11:**
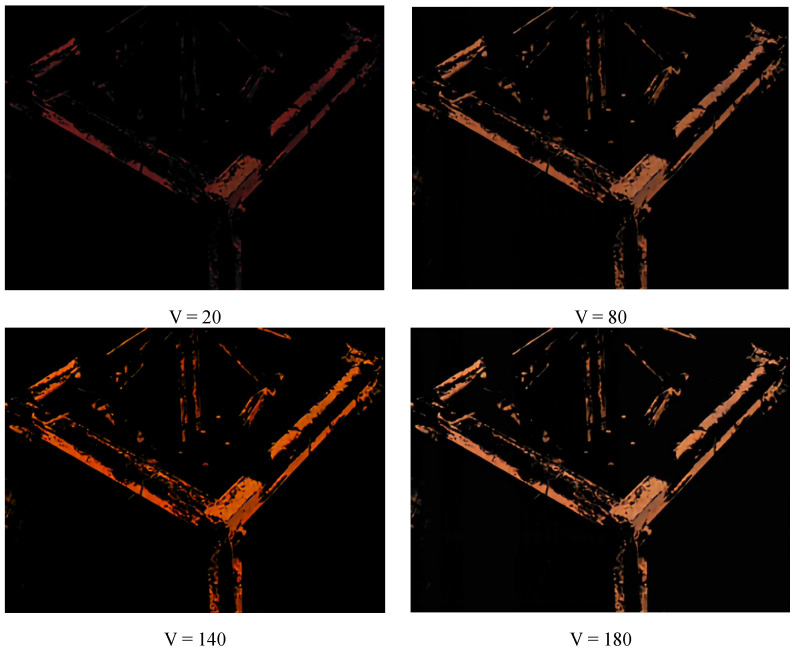
Rust detection results under the same S, H values, and different V values.

The HSV method can be affected by low light and brightness, but the experimental environment is selected to ensure illumination and avoid impact. The values of H, S, and V may affect the rust extraction. Figure 11 shows the rust extraction at different V value, the processed image comes from Figure 10a, the H and S values remain unchanged at 200 and 10, respectively. It is obvious that the rust extraction effect with V = 20 is significantly inferior to the other three situations. It indicates that under conditions outside the threshold range, the rust extraction effect is poor. Compared with Figure 10a, the rust extraction amount with V = 80 is less than those with V = 140 and V = 180. To further analyze the impact of all the parameters in Table 4 on rust detection, a lot of experiments are carried out, and the results are shown in Table 10. It can be seen from the table that under the condition of fixed V value, within the threshold range given in Table 7, changing the values of S and H has little effect on the extraction results of corroded pixels. But when changing the V value, the extracted corroded pixels change significantly. Hence, brightness has a significant impact on the accuracy of rust detection.

**Figure 12:**
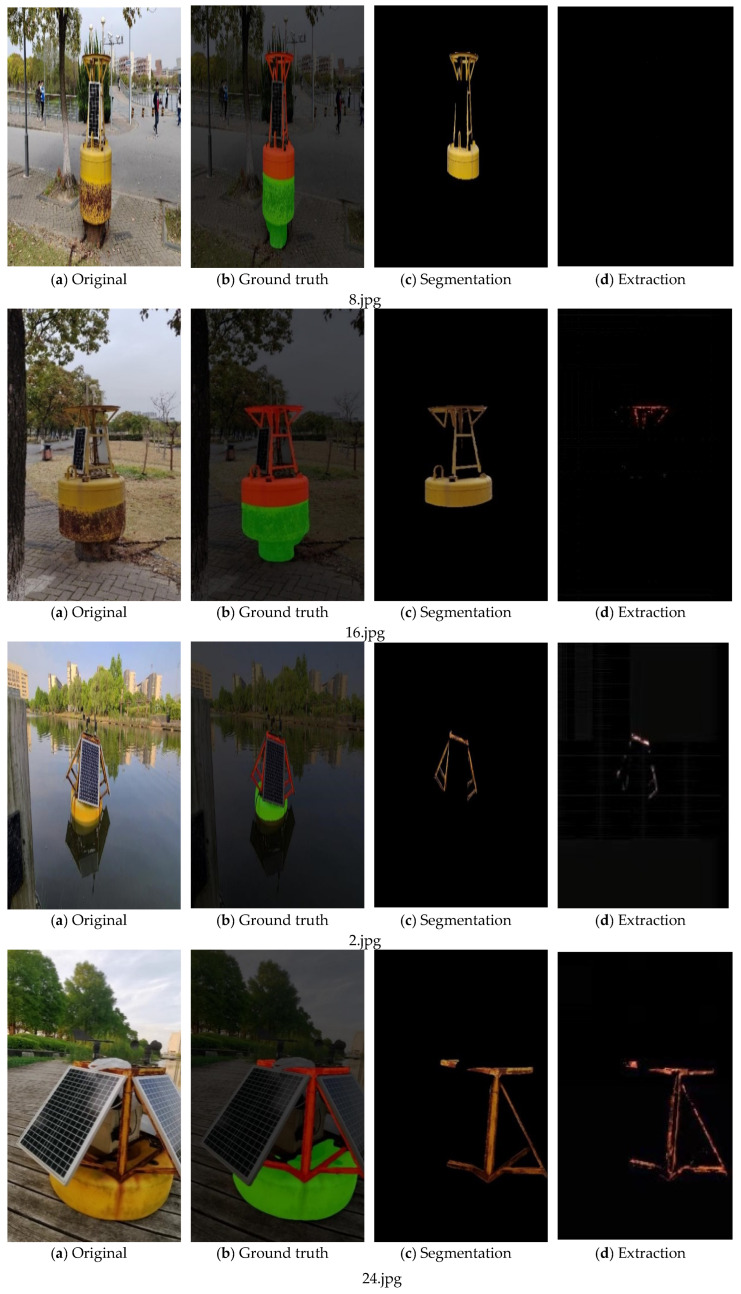
Results of buoy rust extraction by fusing image segmentation and image processing.

As shown in Figure 12, the processing results of the fusion algorithm are (a) for the whole buoy image without obvious rust, where (a) is under good lighting conditions and (b) is under slightly poor backlight conditions; (c) and (d) for the buoy image with obvious rust, where (c) is under good lighting conditions and (d) is taken with slightly poor backlight intensity. The first image of each group is the image to be processed, and two kinds of buoys are chosen in the experiment: buoys onshore and in water. The second one is the result after segmentation by the improved U-Net model, here the red, green, and black zones are the same as those in Figure 10. The third one is the image of extracted metal part of the buoys; the non-metallic parts are filtered. The fourth one is the extraction of the rusted part. After extraction of metal part by color space conversion method, the rusted part is extracted from the metal part.

The improved U-Net model is used to segment the metal part of the buoy image to exclude the influence of the background part and the rest non-metal parts of the beacon on the rust extraction; secondly, the metal part of the beacon image is extracted and the rust of the metal part of the buoy is extracted by using the RGB to HSV color space conversion method. At the same time, the number of pixels in the metal part of the buoy image and the number of pixels in the metal rust part are counted, and the rust level is calculated by Equation (7), and the computer output results are shown below.

As seen in Table 11, 8.jpg rust accounted for about 0.18%, no obvious rust; 16.jpg rust accounted for 0.77%, no obvious rust; 2.jpg rust accounted for 25.0%, obvious rust; and 24.jpg rust accounted for 44.1%, obvious rust existed. The rust grade classification and system output hints in Table 9 are no rust, no rust, moderate rust and severe rust, respectively. It can be seen that different lighting conditions will have some influence on the extraction of rust; the assessment of rust percentage and rust level for the same buoy under different shooting angles will be different, but for the same buoy, the assessment results of rust level using different angles are the same.

In order to better illustrate the effectiveness of this method in extracting the rust, several methods have been introduced as comparative methods (DA Net [21], 2021; Grayscale [33], 2022), shown in Table 12. The data source comes from Figure 12d. It can be seen that the improved U-Net+HSV method performs well in rust extraction. The accuracy of metal segmentation may affect rust extraction.

When creating the dataset, we chose scenes with good lighting, but the effect was limited when the lighting was poor. However, the amount of data is still lacking, which has certain limitations on the accuracy and generalization ability of the model. Next, we will study the extraction of rust under different lighting conditions and increase the rust dataset of the navigation buoy.

## 5. Conclusions

The rust segmentation and extraction method based on improved U-Net and HSV color space is introduced to our work. It has a lower false detection rate compared to the grayscale threshold method.

1. The improved U-Net has excellent segmentation accuracy. Compared to traditional and state-of-the-art methods, the segmentation accuracy of this improved method by 6% to 35% higher. By using data augmentation methods, the problem of limited test datasets is solved. However, due to the construction of a lightweight network, the overall computational load of the network is compressed, and the processing speed is greatly improved;
2. This fusion method can better extract rust areas and provide a more effective reference for rust levels. In the HSV color space, for the rust extraction, the value of v has the greatest impact on the extraction results.

Therefore, based on expanding the navigation mark corrosion dataset, image segmentation methods can be considered to segment the corroded parts in the future.

## Figures and Tables

**Table 1:** Encoder and Decoder details of the proposed network.

**Encoder**						
**Block**	**Filter Size**	**Stride**	**Channels**	**In**	**Out**	**Input**
conv1	7 × 7	2	3/64	S	S/2	Input image
MaxPooling	2 × 2	2	64/64	S/2	S/4	F(conv1)
layer1	3 × 3	1	64/64	S/4	S/4	F(MaxPooling)
layer2	3 × 3	2	64/128	S/4	S/8	F(layer1)
layer3	3 × 3	2	128/256	S/8	S/16	F(layer2)
layer4	3 × 3	2	256/512	S/16	S/32	F(layer3)
**Decoder**						
**Block**	**Filter Size**	**Stride**	**Channels**	**In**	**Out**	**Input**
de4upconv	2 × 2	2	3/64	S/32	S/16	F(layer4)
de4se		1	512/256	S/16	S/16	F(de4upconv ⊗layer3)
de4conv	3 × 3	1	512/256	S/16	S/16	F(de4se)
de3upconv	2 × 2	2	256/128	S/16	S/8	F(de4conv)
de3se		1	256/256	S/8	S/8	F(de3upconv ⊗layer2)
de3conv	3 × 3	1	256/128	S/8	S/8	F(de3se)
de2upconv	2 × 2	2	128/64	S/8	S/4	F(de3conv)
de2se		1	128/128	S/4	S/4	F(de2upconv ⊗layer1)
de2conv	3 × 3	1	128/64	S/4	S/4	F(de2se)
de1upconv	2 × 2	2	64/64	S/4	S/2	F(de2conv)
de1se		1	128/128	S/2	S/2	F(de1upconv ⊗conv1)
de1conv	3 × 3	1	128/64	S/2	S/2	F(de1se)
upconv	3 × 3	2	64/2	S/2	S	F(de1conv)

**Table 2:** Evaluation table of rust grade of the metal part of navigation mark.

Rust Grade	$r_{Rust}$	Brief Description
No rust	<1%	Normal use.
Slight rust	1~10%	There is slight rust on the metal surface.
Moderate rust	10~50%	There is more rust on the metal surface.
Severe rust	50~75%	The surface coating of the metal part basically failed.
Extremely severe rust	>75%	The surface rust is serious, and the metal part is vulnerable.

**Table 3:** Experimental parameter configuration of the splitting algorithm.

Configuration Parameters	Parameter Values	Descriptions
Freeze_Epoch	100	Total number of freeze training rounds
UnFreeze_Epoch	300	Total number of rounds of model training
Batch-size	1	Number of batches
Init_lr	10^−4^	Maximum learning rate of model
weight_decay	0.00005	Attenuation coefficient

**Table 4:** Comparison of evaluation indexes for the performance of different segmentation models.

Model	mPrecision	mRecall	mAcc	mIOU	F1	FPS
U-Net [22]	0.907	0.956	0.945	0.847	0.931	10.5
SegNet [27]	0.832	0.950	0.981	0.804	0.887	20
DeepLabV3+ [28]	0.910	0.942	0.940	0.869	0.925	35
PSPNet [30]	0.906	0.952	0.966	0.884	0.928	20
DANet [21]	0.946	0.964	0.973	0.909	0.959	34
**Ours**	**0.982**	**0.981**	**0.996**	**0.972**	**0.981**	**40**

**Table 5:** Comparison of evaluation metrics of multi-model segmentation performance for a single category (metals).

Model	Precision	Recall	Acc	IOU	F1	FPS
U-Net	0.837	0.851	0.990	0.786	0.843	10
SegNet	0.586	0.899	0.976	0.550	0.709	20
DeepLabV3+	0.782	0.870	0.988	0.700	0.823	35
PSP Net	0.830	0.852	0.986	0.766	0.840	20
DA Net	0.872	0.911	0.993	0.829	0.891	36
**Ours**	**0.942**	**0.947**	**0.996**	**0.882**	**0.937**	**38**

**Table 6:** Comparison of the proposed method with and without data augmentation.

Model	mPrecision	mRecall	mAcc	mIOU
Without Data augmentation	0.950	0.944	0.990	0.907
**Data augmentation**	**0.982**	**0.981**	**0.996**	**0.972**

**Table 7:** HSV color thresholds for rusting materials on metal surfaces.

	H_min_	H_max_	S_min_	S_max_	V_min_	V_max_
Threshold	0	15	140	255	80	180

**Table 8:** Comparison of the pixel values and ratios of the rusted image areas.

	A_1_	A_2_
Number of pixel points of rusted part	19,069	1832
Number of pixel points of the metallic part	24,504	25,517
$r_{rust}$	77.82%	7.17%
Rust grade	Extremely Severe rust	Slight rust

**Table 9:** Comparison of pixels and ratios in two regions of rust images.

	Image (a)	Image (c)
Number of pixel points in the rusted part	511,306	174,523
Number of pixel points of the metal part	700,419	260,093
$r_{rust}$	73.1%	67.1%
Rust grade	Severe rust	Severe rust

**Table 10:** The extraction of pixel points of the rust under different H, S, V values.

U	S	V	Number of Pixel Points
0	200	140	510,897
8	200	140	511,306
15	200	140	510,376
8	140	140	510,087
8	180	140	510,213
8	255	140	500,812
8	200	80	497,342
8	200	180	511,278

**Table 11:** Results of buoy rust extraction by fusing image segmentation and image processing.

Image Name	8	16	2	24
Number of Pixels of rust	128	857	4720	40,791
Number of Pixels of metal	73,036	111,795	18,876	92,463
$r_{rust}$	1.75%	0.76%	25%	44.11%
Rust grade	None	None	Moderate corrosion	Moderate corrosion

**Table 12:** Comparison of the rust extraction under different methods.

Method	Number of Pixels of Rust	Method	Number of Pixels of Rust
Ours	40,791	Improve Net+Grayscale [32]	39,407
DA Net [21] + HSV	40,236	U-Net+RGB	39,143

## Data Availability

Access to the data will be considered upon request by the authors.
